# Health outcomes and care needs after osteoporotic fractures in rural Chinese older adults: policy implications

**DOI:** 10.3389/fpubh.2025.1601892

**Published:** 2025-06-25

**Authors:** Qian Zhu, Caixia Ran

**Affiliations:** Department of Orthopedics and Traumatology, The Central Hospital of Enshi Tujia and Miao Autonomous, Hubei, China

**Keywords:** osteoporotic fractures, older adult care, rural China, socioeconomic disparities, policy awareness, family support

## Abstract

**Background:**

Osteoporotic fractures pose a significant public health challenge among the older adult in rural settings with limited healthcare access. This study investigated the burden of osteoporotic fractures, associated care needs, and influencing factors in rural China.

**Methodology:**

A cross-sectional study was conducted from March 2022 to December 2024, involving older adult individuals aged ≥60 years from rural regions of Enshi Prefecture, Hubei Province, as well as Shandong, Henan, Yunnan, and Gansu provinces in China. Participants were recruited via community health centers, with eligibility confirmed through medical records for osteoporosis or osteoporotic fracture history. A validated 33-item questionnaire assessed demographics, family support, health status, healthcare access, and policy awareness, with logistic regression analyzing factors associated with receiving help after fractures, adjusting for confounders.

**Results:**

Among a total of 3,600 participants, 58.7% reported osteoporotic fractures, with 50.2% experiencing life impact, strongly linked to recent falls (93.2%, *p* < 0.001). Socioeconomic disparities were evident, with insured individuals (OR 2.18, 95% CI 1.95–2.40, *p* < 0.001) and homeowners (OR 2.65, 95% CI 2.40–2.90, *p* < 0.001) more likely to receive help after fractures. Low policy awareness — defined as < 3 correct answers on a (0–6) Rural Health-Policy Knowledge Index — (OR 0.22, 95% CI 0.15–0.30, *p* < 0.001), and a high need for government support (90.1%) highlighted barriers to care. Medical interventions, including supplement use (OR 5.07, 95% CI 4.80–5.35, *p* < 0.001) and osteoporosis treatment (OR 4.51, 95% CI 4.32–4.73, *p* < 0.001), were significantly associated with increased odds of receiving help following osteoporotic fractures. Family support dynamics showed variability, with children helping after fracture reducing formal care access (OR 0.79, 95% CI 0.65–0.95, *p* = 0.013).

**Conclusion:**

Osteoporotic fractures impose a substantial burden on rural Chinese older adult, exacerbated by socioeconomic disparities and low policy awareness. Enhancing insurance coverage, health education, and access to medical interventions is critical to address care inequities and improve outcomes.

## 1 Introduction

Osteoporotic fractures, arising from compromised bone density and microstructural integrity, pose a formidable challenge to global public health, particularly among aging cohorts (1, 2). Individuals aged over 60 years face elevated risks owing to progressive bone loss and declining physical capacity, with fractures precipitating substantial morbidity, mortality, and healthcare expenditure (3). In rural regions, limited healthcare access, socioeconomic disadvantage, and dependence on informal caregiving amplify this burden (4). Although osteoporosis afflicts an estimated 200 million individuals worldwide, the interconnections among fracture occurrence, subsequent health outcomes, and resultant care demands remain inadequately characterized, especially within resource-scarce rural contexts (2, 5, 6). Notably, while hip fractures predominate in disability metrics, the broader spectrum of fragility fractures and their caregiving ramifications warrants further investigation (7, 8).

Extant literature delineates the clinical and fiscal implications of osteoporosis (9); however, the influence of social determinants—such as familial assistance, financial sufficiency, and healthcare accessibility—on post-fracture trajectories remains insufficiently explored (10). Equally, the extent to which older adults perceive skeletal fragility, pursue therapeutic interventions, and rely on kinship networks for support constitutes a significant evidentiary void (11, 12). This lacuna assumes critical importance as the global demographic aged 60 years and older is projected to constitute 22% of the population by 2050 (13, 14). Absent rigorous data to inform intervention strategies, the escalating prevalence of osteoporotic fractures risks overwhelming informal care frameworks and straining public health systems, thereby entrenching cycles of disability and socioeconomic disparity (15, 16). Furthermore, the paucity of evidence regarding osteoporosis awareness and preventive engagement in rural populations impedes the formulation of efficacious public health measures (17, 18).

Current study seeks to redress critical gaps in the extant literature by rigorously examining the associations between osteoporotic fractures, clinical outcomes, and caregiving demands in a rural context. Such an analysis is essential for the development of evidence-based strategies to prevent osteoporosis, optimise post-fracture management, and strengthen community-based support systems, thereby attenuating the societal and economic sequelae of skeletal fragility in ageing populations (19–21). The inclusion of patient-reported outcomes—namely, perceptions of bone health and fracture-related concerns—enhances the capacity to assess both the clinical manifestations and psychosocial ramifications of this condition (21, 22).

In the People’s Republic of China, rapid demographic aging—with over 264 million individuals aged 60 years and older in 2020, projected to reach 402 million by 2040—exacerbates the osteoporosis burden, with rural regions disproportionately impacted by healthcare disparities (23, 24). Prevalence estimates suggest that 20 to 30% of older Chinese adults are affected, yet data specific to rural fracture outcomes and care dependencies remain limited. These disparities are compounded by constrained insurance coverage and healthcare access, placing additional pressure on traditional familial care systems (25, 26). The burden of osteoporotic fractures in rural China remains underexplored, particularly regarding socioeconomic factors, family support, and policy awareness in shaping care access. This study aimed to investigate the prevalence and life impact of osteoporotic fractures among rural older adult, identify barriers to care, and evaluate the role of family support and medical interventions in addressing care needs, providing insights for public health interventions to reduce fracture-related morbidity in rural settings.

## 2 Methodology

### 2.1 Study design

The current cross-sectional study was conducted from March 2022 to December 2024, involving older adult individuals aged ≥60 years from rural regions of Enshi Prefecture, Hubei Province, China, as well as from Shandong, Henan, Yunnan, and Gansu provinces. Although not geographically adjacent to Enshi, Shandong was included to enhance regional representation by capturing variation in healthcare access, socioeconomic conditions, and demographic profiles across eastern and central-southwestern rural China. A structured questionnaire was administered to collect data on demographic characteristics, family and social support, health and osteoporosis status, healthcare access, and policy awareness among the target population. The cross-sectional approach was chosen to capture the prevalence of osteoporotic fractures and associated factors, such as the likelihood of receiving help after a fracture, and to explore the impact of these fractures on daily life, which were key outcomes of the study. This design facilitated the identification of associations between variables like osteoporosis treatment, family support, and healthcare access, which were central to the study’s objectives of understanding older adult care needs in a rural context. These provinces were selected based on prior national epidemiologic data indicating high prevalence of osteoporosis combined with limited healthcare infrastructure, enabling assessment of care gaps in high-risk, underserved older adult populations.

### 2.2 Study population

We recruited 3,600 community-dwelling individuals aged ≥60 years from rural areas across five Chinese provinces—Enshi Prefecture (Hubei), Shandong, Henan, Yunnan, and Gansu—between March 2022 and December 2024. Participants were eligible regardless of osteoporosis status or fracture history to ensure a representative sample for assessing older adult care needs across varying health risk profiles. Recruitment was conducted via township-level community health centers, using medical records for eligibility screening. The Central Hospital of Enshi Prefecture served as a coordinating site during early recruitment.

The sample size was calculated based on an estimated 18.9% prevalence of osteoporotic fractures among older adult individuals in China, as reported in a recent systematic review and meta-analysis (27). with the formula *n = Z^2^ × p*(*1–p*) */d^2^* (*Z* = 1.96, *p* = 0.189, d = 0.05), yielding *n₀* = 2,367. After adjusting for a 10% non-response rate (*n₁* = 2,620) and a design effect of 1.4 due to provincial stratification, the final target was 3,668. A total of 3,600 valid responses were obtained (response rate = 98.1%), ensuring sufficient power for subgroup and multivariable analyses of post-fracture care outcomes.

### 2.3 Inclusion and exclusion criteria

Eligible participants were aged ≥60 years, rural residents for ≥12 months, with either a confirmed osteoporosis diagnosis (BMD T-score ≤ − 2.5 at lumbar spine, femoral neck, or total hip via DXA, per WHO criteria) or a history of low-trauma fracture after age 50 (verified by medical records or radiographic evidence as a proxy for osteoporosis). This allowed inclusion of individuals at risk of osteoporotic fractures due to osteoporosis, even if they had not yet experienced a fracture, ensuring a comprehensive assessment of care needs in this population. Participants required a Mini-Mental State Examination (MMSE) score ≥24 for reliable responses and provided written consent. Exclusions included urban residents, recent rural migrants (<12 months), individuals with secondary osteoporosis (e.g., due to hyperthyroidism or long-term corticosteroid use), severe comorbidities (e.g., advanced cancer, end-stage renal disease, major neurological disorders), MMSE score <24, or unwillingness to consent. These criteria were applied to ensure a more homogeneous older adult rural population and reduce variability in functional status and underlying health conditions that could bias associations with post-fracture care outcomes.

### 2.4 Questionnaire development

The questionnaire was developed rigorously to ensure validity, reliability, and cultural relevance for rural Chinese older adult at risk of osteoporotic fractures. A literature review identified key domains—demographics, family support, health and osteoporosis status, healthcare access, and policy awareness—based on studies from China and low-to middle-income countries. A panel of geriatricians, epidemiologists, public health researchers, and sociologists refined these domains, ensuring relevance to rural China, including factors like family reliance and government support needs. The questionnaire comprised five structured domains. The first section assessed demographics, including age, sex, residential setting, and home ownership. The second section focused on family support, capturing co-residence with children, financial dependence, and assistance received following fracture. “Receiving help after fracture” was operationalized as a binary (yes/no) variable based on participant self-report of any physical, emotional, or financial assistance received from family members, neighbors, or caregivers during the post-fracture recovery period. The third section covered health and osteoporosis status, including history of fractures (both osteoporotic and non-osteoporotic), formal diagnosis of osteoporosis by a physician, past falls, perceived skeletal fragility, and limitations in physical activity.

The fourth section addressed healthcare access and resources. Income was assessed through self-reported total monthly household income, categorized into low, middle, or high levels based on national rural poverty thresholds and local cost-of-living benchmarks. Participants were also asked whether they perceived their income to be sufficient for meeting basic living and healthcare needs; this was recorded as a binary variable (“income sufficiency”: yes/no). Other variables in this section included insurance coverage, healthcare service utilization, and use of mobility aids. Two binary items captured osteoporosis-related medical interventions: (1) current use of calcium and/or vitamin D supplements, and (2) receipt of pharmacologic treatment (e.g., bisphosphonates or calcitonin). Given the rural healthcare context—where access to pharmacologic therapies is limited—supplements are often used as first-line or sole treatment; therefore, these variables were analyzed independently to reflect their distinct implications for care.

The fifth section assessed knowledge and policy awareness through six binary-response items querying awareness of public entitlements such as medication subsidies, access to fracture rehabilitation, and home-based eldercare programs. Each correct response was scored as one point (total range: 0–6), and scores below 3 were classified as ‘low policy awareness,’ based on pilot testing and expert consensus. Additionally, participants were asked whether their most recent fracture had a significant impact on daily functioning, specifically regarding mobility, ability to perform basic tasks (e.g., walking, cooking, toileting), and level of dependence on others. Responses were recorded as a binary variable (“life impact”), reflecting the participant’s subjective perception of lasting disruption to independent living. All questionnaire items were administered using binary, multiple-choice, or 5-point Likert-scale formats to accommodate low literacy levels common among rural older adult respondents.

The draft was pilot-tested with 50 older adult individuals, with feedback leading to simplified terms (e.g., “osteoporotic fracture” to “bone break due to weak bones”) for better comprehension. Validation involved content validity (expert panel) and construct validity (exploratory factor analysis), with internal consistency (Cronbach’s alpha: 0.82) and test–retest reliability (correlation coefficient: 0.87, *n* = 30, 2 weeks apart) confirming reliability. The final questionnaire comprised 33 items and was designed to be completed in 20–30 min (approximately 1–2 questions per minute, accounting for literacy challenges). It was initially developed in English, translated into Mandarin, and then back-translated into English by a multidisciplinary team including bilingual public health researchers, native speakers of Tujia and Miao dialects, and a certified medical translator. Cultural and linguistic adaptations were made to ensure clarity and appropriateness for older adult respondents in Enshi Prefecture and other rural regions.

### 2.5 Data collection technique and procedure

Participants were recruited from rural areas across five provinces—Enshi Prefecture (Hubei), Shandong, Henan, Yunnan, and Gansu—through township-level community health centers, using medical records to confirm eligibility. Trained research assistants fluent in Mandarin and relevant local dialects (e.g., Tujia, Miao) conducted home visits and community-based assessments to maximize participation and represent regional diversity. Sex-stratified sampling ensured adequate representation for analyses of gender-based differences in outcomes, such as help received after fracture. To address literacy and sociolinguistic barriers, questionnaires were orally administered when needed, supported by visual aids and culturally adapted scripts. Verbal informed consent was obtained from all participants. Data were collected on paper, digitized into a secure database, and 10% were randomly double-entered for quality control, with discrepancies resolved using original forms to ensure the accuracy of key variables, including daily task assistance, care needs, and post-fracture support.

Rural residency was defined based on household registration (hukou) and self-reported residence in a rural or peri-urban area for at least 12 months prior to the survey. Participants residing in township areas but registered under rural hukou were retained due to shared exposure to rural healthcare and socioeconomic conditions.

### 2.6 Statistical analysis

Analysis was performed using R software (version 4.3.1). Descriptive statistics were used to summarize the sample, means and standard deviations were reported for continuous variables (e.g., age), and frequencies and percentages for categorical variables (e.g., sex, osteoporosis diagnosis, supplement use). Group differences (e.g., receiving help after fracture vs. not) were evaluated using chi-square tests for categorical variables and independent *t*-tests for continuous variables.

Multivariable logistic regression was used to estimate adjusted odds ratios (ORs) with 95% confidence intervals for factors associated with receiving help after fracture. Key predictors—such as osteoporosis treatment and supplement use—were entered simultaneously into the model along with covariates including age, sex, insurance status, income sufficiency, activity limitation, and region. Variance inflation factors (VIF) were assessed to check for multicollinearity between variables, particularly between supplement use and treatment, and no issues were identified. A two-sided *p*-value <0.05 was considered statistically significant.

## 3 Results

The study cohort comprised 3,600 older adult participants from rural China, with a mean age of 66.5 years (SD 7.9) and a balanced sex distribution (50.1% female). Table 1 summarizes the baseline characteristics. Socioeconomically, 80.0% were rural residents, 50.1% were homeowners, and 63.9% reported sufficient income. Family support was prevalent, with 87.2% having children, 80.5% living with children, and 70.1% relying financially on family. Health status revealed 73.0% with a fracture history, 58.7% with an osteoporotic fracture, and 40.0% with a confirmed osteoporosis diagnosis, of whom 40.1% were receiving treatment. Additionally, 53.8% of participants reported experiencing a recent fall, 53.6% perceived their bones as weak, and 60.1% expressed concern about sustaining a fracture. Healthcare access was limited, with 54.8% reporting access, 53.7% insured, and 40.1% using supplements; 20.2% used mobility aids. Policy awareness was moderate, with 61.3% having osteoporosis knowledge, 49.9% aware of relevant policies, and 90.1% reporting a need for government support.

**Table 1:** Characteristics of study participants in rural China (*N* = 3,600).

Variable	Value
Demographic characteristics	
Age, y, mean ± SD	66.5 ± 7.9
Female, *n* (%)	1,835 (50.1)
Rural resident, *n* (%)	2,929 (80.0)
Home owner, *n* (%)	1,833 (50.1)
Family and social support	
Has children, *n* (%)	3,192 (87.2)
Lives with children, *n* (%)	2,948 (80.5)
Family financial reliance, *n* (%)	2,564 (70.1)
Family trust, *n* (%)	3,186 (87.0)
Children helped after fracture, *n* (%)	1,470 (40.2)
Received help after fracture, *n* (%)	1,471 (40.2)
Daily task help, *n* (%)	1,108 (30.3)
Health and osteoporosis status	
Fracture history, *n* (%)	2,672 (73.0)
Osteoporotic fracture, *n* (%)	2,148 (58.7)
Osteoporosis diagnosis, *n* (%)	1,465 (40.0)
Osteoporosis treatment, *n* (%)	1,469 (40.1)
Recent fall, *n* (%)	1,970 (53.8)
Perceived bone weakness, *n* (%)	1,963 (53.6)
Activity avoidance, *n* (%)	2,227 (60.8)
Fracture worry, *n* (%)	2,199 (60.1)
Life impact, *n* (%)	1,837 (50.2)
Healthcare and resources	
Sufficient income, *n* (%)	2,337 (63.9)
Insured, *n* (%)	1,966 (53.7)
Healthcare access, *n* (%)	2,004 (54.8)
Medical treatment, *n* (%)	2,199 (60.1)
Supplement use, *n* (%)	1,469 (40.1)
Mobility aid, *n* (%)	739 (20.2)
Care difficulty, *n* (%)	1,108 (30.3)
Health satisfaction, *n* (%)	2,198 (60.1)
Knowledge and policy awareness	
Osteoporosis knowledge, *n* (%)	2,244 (61.3)
Policy awareness, *n* (%)	1,827 (49.9)
Policy care benefit, *n* (%)	2,926 (79.9)
Policy safety perception, *n* (%)	1,834 (50.1)
Government support need, *n* (%)	3,296 (90.1)

Table 2 presents characteristics stratified by key outcomes: receiving help after fracture, life impact, fracture history, and osteoporosis treatment. Of the participants, 40.2% received help after a fracture, with significant differences by sex (48.8% females vs. 51.2% males, *p* < 0.001), insurance status (61.4% insured vs. 38.6% uninsured, *p* < 0.001), and treatment status (78.6% treated vs. 21.4% untreated, *p* < 0.001). Homeownership was associated with receiving help (65.8% homeowners vs. 34.2% non-homeowners, *p* < 0.001). Life impact was reported by 50.2%, strongly linked to osteoporotic fractures (85.5% with life impact vs. 14.5% without, *p* < 0.001) and recent falls (93.2% vs. 6.8%, *p* < 0.001). Fracture history was reported by 73.0%, with females more affected (81.0% vs. 19.0% males, *p* < 0.001) and rural residents showing higher prevalence (71.0% vs. 29.0% non-rural, *p* < 0.001). Osteoporosis treatment (40.1%) was more common among females (60.2% vs. 39.8% males, *p* < 0.001) and insured individuals (74.7% vs. 25.3% uninsured, *p* < 0.001).

**Table 2:** Characteristics of study participants by received help after fracture, life impact, fracture history, and osteoporosis treatment in rural China (*N* = 3,600).

Variable	Received help after fracture			Life impact			Fracture history			Osteoporosis treatment		
	No (*n* = 2,189)	Yes (*n* = 1,471)	*p* Value	No (*n* = 1,823)	Yes (*n* = 1,837)	*p* Value	No (*n* = 988)	Yes (*n* = 2,672)	*p* Value	No (*n* = 2,191)	Yes (*n* = 1,469)	*p* Value
Demographic characteristics												
Age, y, mean ± SD	66.5 ± 9.3	66.4 ± 5.1	0.044	66.8 ± 9.1	66.2 ± 6.4	0.036	71.1 ± 9.7	64.8 ± 6.3	<0.001	66.6 ± 10.0	66.3 ± 2.2	0.8
Female, *n* (%)	940 (51.2)	895 (48.8)	<0.001	1,096 (59.7)	739 (40.3)	<0.001	349 (19.0)	1,486 (81.0)	<0.001	731 (39.8)	1,104 (60.2)	<0.001
Rural resident, *n* (%)	1,564 (53.4)	1,365 (46.6)	<0.001	1,459 (49.8)	1,470 (50.2)	>0.9	849 (29.0)	2,080 (71.0)	<0.001	1,460 (49.8)	1,469 (50.2)	<0.001
Home owner, *n* (%)	626 (34.2)	1,207 (65.8)	<0.001	1,461 (79.7)	372 (20.3)	<0.001	348 (19.0)	1,485 (81.0)	<0.001	364 (19.9)	1,469 (80.1)	<0.001
Family and social support												
Has children, *n* (%)	1,831 (57.4)	1,361 (42.6)	<0.001	1,450 (45.4)	1,742 (54.6)	<0.001	900 (28.2)	2,292 (71.8)	<0.001	1,799 (56.4)	1,393 (43.6)	<0.001
Lives with children, *n* (%)	1,649 (55.9)	1,299 (44.1)	<0.001	1,309 (44.4)	1,639 (55.6)	<0.001	633 (21.5)	2,315 (78.5)	<0.001	1,638 (55.6)	1,310 (44.4)	<0.001
Family financial reliance, *n* (%)	1,564 (61.0)	1,000 (39.0)	0.025	1,096 (42.7)	1,468 (57.3)	<0.001	487 (19.0)	2,077 (81.0)	<0.001	1,460 (56.9)	1,104 (43.1)	<0.001
Family trust, *n* (%)	1,821 (57.2)	1,365 (42.8)	<0.001	1,414 (44.4)	1,772 (55.6)	<0.001	606 (19.0)	2,580 (81.0)	<0.001	1,770 (55.6)	1,416 (44.4)	<0.001
Children helped, *n* (%)	939 (63.9)	531 (36.1)	<0.001	888 (60.4)	583 (39.6)	<0.001	573 (39.0)	897 (61.0)	<0.001	317 (21.5)	1,154 (78.5)	<0.001
Daily task help, *n* (%)	630 (56.9)	478 (43.1)	0.016	362 (24.6)	1,108 (75.4)	<0.001	211 (19.0)	897 (81.0)	<0.001	1,098 (74.7)	372 (25.3)	<0.001
Health and osteoporosis status												
Fracture history, *n* (%)	1,628 (60.9)	1,044 (39.1)	0.023	1,184 (44.3)	1,488 (55.7)	<0.001	—	—	—	1,482 (55.5)	1,190 (44.5)	<0.001
Osteoporotic fracture, *n* (%)	1,440 (67.0)	708 (33.0)	<0.001	311 (14.5)	1,837 (85.5)	<0.001	375 (19.0)	1,595 (81.0)	<0.001	1,589 (74.0)	559 (26.0)	<0.001
Osteoporosis diagnosis, *n* (%)	624 (42.6)	841 (57.4)	<0.001	1,097 (59.8)	737 (40.2)	<0.001	278 (19.0)	1,187 (81.0)	<0.001	365 (19.9)	1,469 (80.1)	<0.001
Osteoporosis treatment, *n* (%)	315 (21.4)	1,154 (78.6)	<0.001	364 (24.8)	1,101 (75.2)	<0.001	279 (19.0)	1,190 (81.0)	<0.001	—	—	—
Recent fall, *n* (%)	1,333 (67.7)	637 (32.3)	<0.001	133 (6.8)	1,837 (93.2)	<0.001	580 (29.5)	1,383 (70.5)	<0.001	1,517 (77.0)	453 (23.0)	<0.001
Perceived bone weakness, *n* (%)	1,630 (83.0)	333 (17.0)	<0.001	507 (25.8)	1,456 (74.2)	<0.001	442 (19.7)	1,802 (80.3)	<0.001	1,806 (92.0)	157 (8.0)	<0.001
Activity avoidance, *n* (%)	1,020 (45.8)	1,207 (54.2)	<0.001	1,253 (56.3)	974 (43.7)	<0.001	426 (19.1)	1,801 (80.9)	<0.001	758 (34.0)	1,469 (66.0)	<0.001
Fracture worry, *n* (%)	1,563 (71.1)	636 (28.9)	<0.001	362 (16.5)	1,837 (83.5)	<0.001	711 (32.3)	1,488 (67.7)	<0.001	1,827 (83.1)	372 (16.9)	<0.001
Life impact, *n* (%)	1,254 (68.3)	583 (31.7)	<0.001	—	—	—	349 (19.0)	1,488 (81.0)	<0.001	1,465 (79.7)	372 (20.3)	<0.001
Healthcare and resources												
Sufficient income, *n* (%)	1,554 (66.5)	783 (33.5)	<0.001	500 (21.4)	1,837 (78.6)	<0.001	448 (19.2)	1,889 (80.8)	<0.001	1,665 (71.2)	672 (28.8)	<0.001
Insured, *n* (%)	759 (38.6)	1,207 (61.4)	<0.001	1,487 (75.6)	479 (24.4)	<0.001	374 (19.0)	1,592 (81.0)	<0.001	497 (25.3)	1,469 (74.7)	<0.001
Healthcare access, *n* (%)	1,634 (81.5)	370 (18.5)	<0.001	503 (25.1)	1,501 (74.9)	<0.001	640 (31.9)	1,364 (68.1)	<0.001	1,863 (93.0)	141 (7.0)	<0.001
Medical treatment, *n* (%)	1,563 (71.1)	636 (28.9)	<0.001	0 (0.0)	1,108 (100.0)	<0.001	711 (32.3)	1,488 (67.7)	<0.001	736 (66.4)	372 (33.6)	<0.001
Supplement use, *n* (%)	315 (21.4)	1,154 (78.6)	<0.001	1,097 (74.7)	372 (25.3)	<0.001	427 (29.0)	1,044 (71.0)	0.023	0 (0.0)	1,469 (100.0)	<0.001
Mobility aid, *n* (%)	314 (42.5)	425 (57.5)	<0.001	0 (0.0)	1,108 (100.0)	<0.001	141 (19.1)	598 (80.9)	<0.001	736 (66.4)	372 (33.6)	<0.001
Care difficulty, *n* (%)	630 (56.9)	478 (43.1)	0.016	362 (16.5)	1,837 (83.5)	<0.001	211 (19.0)	897 (81.0)	<0.001	1,827 (83.1)	372 (16.9)	<0.001
Health satisfaction, *n* (%)	938 (42.7)	1,260 (57.3)	<0.001	1,461 (66.5)	737 (33.5)	<0.001	417 (19.0)	1,781 (81.0)	<0.001	729 (33.2)	1,469 (66.8)	<0.001
Knowledge and policy awareness												
Osteoporosis knowledge, *n* (%)	1,280 (57.0)	964 (43.0)	<0.001	407 (18.1)	1,837 (81.9)	<0.001	279 (19.0)	1,190 (81.0)	<0.001	1,487 (66.3)	757 (33.7)	<0.001
Policy awareness, *n* (%)	1,563 (85.6)	264 (14.4)	<0.001	0 (0.0)	739 (100.0)	<0.001	640 (35.0)	1,187 (65.0)	<0.001	367 (49.7)	372 (50.3)	<0.001
Policy care benefit, *n* (%)	1,873 (64.0)	1,053 (36.0)	<0.001	362 (19.8)	1,465 (80.2)	<0.001	849 (29.0)	2,077 (71.0)	<0.001	1,827 (100.0)	0 (0.0)	<0.001
Policy safety perception, *n* (%)	627 (34.2)	1,207 (65.8)	<0.001	1,458 (49.8)	1,468 (50.2)	>0.9	348 (19.0)	1,486 (81.0)	<0.001	1,822 (62.3)	1,104 (37.7)	<0.001
Government support need, *n* (%)	1,878 (57.0)	1,418 (43.0)	<0.001	1,459 (44.3)	1,837 (55.7)	<0.001	919 (27.9)	2,377 (72.1)	<0.001	1,827 (55.4)	1,469 (44.6)	<0.001

**Figure 1:**
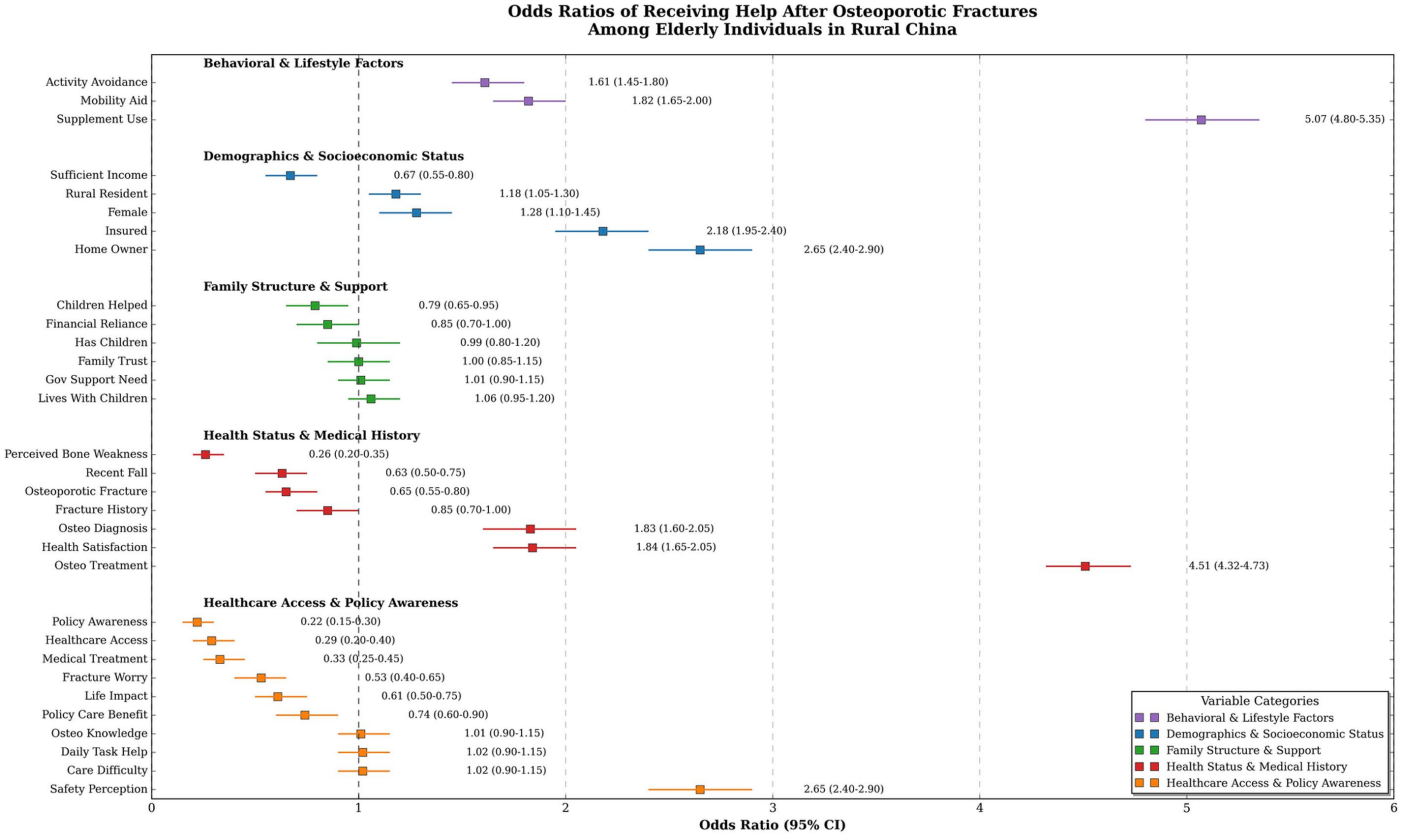
Odds ratios for receiving help after osteoporotic fractures in Rural Enshi Prefecture, Hubei, China. Odds ratios (OR) with 95% confidence intervals (CI) for factors associated with receiving help after osteoporotic fractures, categorized by behavioral (purple), demographic (blue), family (green), health (red), and policy (yellow) factors; the dashed line at OR = 1 indicates no effect, with points to the right/left showing increased/decreased odds. OR, odds ratio; CI, confidence interval; GOV, government.

Figure 1 displays odds ratios (OR) with 95% confidence intervals (CI) for factors associated with receiving help after osteoporotic fractures. Both supplement use (OR 5.07, 95% CI 4.80–5.35, *p* < 0.001) and osteoporosis treatment (OR 4.51, 95% CI 4.32–4.73, *p* < 0.001) were strongly associated with increased likelihood of receiving assistance. Socioeconomic factors also influenced outcomes, with homeownership (OR 2.65, 95% CI 2.40–2.90, *p* < 0.001) and insurance (OR 2.18, 95% CI 1.95–2.40, *p* < 0.001) significantly increasing the likelihood of receiving help. Conversely, perceived bone weakness (OR 0.26, 95% CI 0.20–0.35, *p* < 0.001) and low policy awareness (OR 0.22, 95% CI 0.15–0.30, *p* < 0.001) were associated with reduced odds of receiving help. Family support dynamics showed variability, with children helping after fracture linked to lower odds (OR 0.79, 95% CI 0.65–0.95, *p* = 0.013), while living with children had no significant effect (OR 1.06, 95% CI 0.95–1.20, *p* = 0.275).

These findings highlight critical public health challenges in rural China. The high prevalence of osteoporotic fractures and their association with life impact and recent falls underscore the significant burden on older adult populations. Socioeconomic disparities, particularly in insurance and homeownership, strongly influence access to care, with insured and homeowner participants more likely to receive help (*p* < 0.001). The limited policy awareness and high need for government support (90.1%) indicate deficiencies in health education and support systems, while the protective effect of medical interventions (supplement use, treatment) suggests potential avenues for improving care outcomes.

## 4 Discussion

This cross-sectional study of 3,600 older adult individuals in rural China provides critical insights into the burden of osteoporotic fractures and associated care needs, revealing a high prevalence of osteoporotic fractures (58.7%), significant life impact (50.2%), and a strong association with recent falls (93.2%, *p* < 0.001). Additionally, the study highlights socioeconomic disparities in care access, with insurance and homeownership significantly increasing the odds of receiving help after fractures, alongside notable barriers such as low policy awareness (OR 0.22, 95% CI 0.15–0.30, *p* < 0.001) and a high need for government support (90.1%). The prevalence of osteoporotic fractures in our cohort is substantially higher than that reported in a systematic review of older adult Chinese populations, which estimated a prevalence of 18.9% among those aged ≥60 years (28). The discrepancy may be attributed to our study’s focus on rural areas, where limited access to preventive care and diagnostic tools like dual-energy X-ray absorptiometry (DXA) may result in underdiagnosis of osteoporosis until a fracture occurs, a challenge also noted in other rural Asian settings (29–31). In contrast, a study in urban Shanghai reported a lower prevalence of osteoporotic vertebral fractures (15.3%) among community-dwelling older adult, likely due to better healthcare access and awareness in urban settings (32). This urban–rural disparity underscores the need for targeted interventions in rural China to address diagnostic and preventive gaps.

Socioeconomic factors significantly influenced care access in our study, with insured participants (OR 2.18, 95% CI 1.95–2.40, *p* < 0.001) and homeowners (OR 2.65, 95% CI 2.40–2.90, *p* < 0.001) more likely to receive help after fractures. This finding aligns with a nationwide study in China, which found that insurance coverage was a key determinant of post-fracture care among older adult patients (33). However, our results contrast with a study in Taiwan, where socioeconomic status had a weaker association with care access (OR 1.40, 95% CI 1.20–1.65), possibly due to Taiwan’s universal healthcare system, which mitigates financial barriers to care (34). The stronger association in rural China may reflect the region’s limited healthcare infrastructure, where insurance and financial stability are critical for accessing even basic care, highlighting a pressing need for health equity initiatives (35–39).

Policy awareness was a significant barrier to care in our study, with low awareness (OR 0.22, 95% CI 0.15–0.30, *p* < 0.001) and a high need for government support (90.1%) among participants. This finding is consistent with a cross-sectional study in Jiangsu Province, China, which reported that only 45.6% of older adult osteoporotic fracture patients were aware of relevant health policies, attributing this to inadequate health education in rural areas (40). In contrast, a study in Hong Kong found higher policy awareness (72.3%) among older adult individuals, likely due to more robust public health campaigns and better healthcare access (41). The lower awareness in our rural cohort may be due to limited outreach and education programs, compounded by lower literacy levels, necessitating tailored interventions to improve health literacy and policy engagement in rural China (26, 42, 43).

Medical interventions, and supplement use both were strongly associated with receiving help after fractures, a finding that aligns with a nationwide study in China showing that anti-osteoporosis medication use significantly improved post-fracture outcomes (44). However, our treatment rate (40.1%) is lower than that reported in a European cohort (68.3%), where universal healthcare ensures broader access to such interventions (9). This disparity may be attributed to the high cost of osteoporosis medications in China and limited insurance coverage in rural areas, which restrict access to treatment (45–47). These findings suggest that improving access to affordable medical interventions could substantially enhance care outcomes in rural China.

The high self-reported need for government support (90.1%) within our population also highlights gaps in health policy and education regarding osteoporosis (48, 49). Many respondents exhibited moderate awareness of relevant policies (49.9%), indicating an urgent need for community-based educational programs tailored to increase awareness of osteoporosis management among elders (49, 50). This correlates with reports from various regions highlighting the effectiveness of education in reducing fracture incidences through improved health literacy (51, 52). However, in terms of contrasting results, our findings show a robust link between homeownership and the likelihood of receiving help (65.8% of homeowners received help), whereas previous studies in urban settings found negligible correlation between living arrangements and support received, where formal support systems were more prevalent (53–55). This difference may suggest varying coping mechanisms and resource availability in rural versus urban older adult populations, necessitating targeted interventions to cater to the unique needs of these demographics.

Interestingly, the data on perceived bone weakness and its negative association with receiving help suggests a potential psychological barrier affecting patient care-seeking behavior. Previous studies have indicated that fear of falls or further injury among older adult individuals often deters them from seeking necessary assistance after fractures (56, 57). This psychological factor could explain why those perceiving increased vulnerability due to osteoporosis were less likely to request or receive support after their fractures.

Family support dynamics in our study revealed that children helping after fracture reduced the odds of receiving formal help (OR 0.79, 95% CI 0.65–0.95, *p* = 0.013), while living with children had no significant effect (OR 1.06, 95% CI 0.95–1.20, *p* = 0.275). This pattern is similar to findings in previous studies, reported older adult Chinese in mainland China, which reported that reliance on family support decreased formal care-seeking, reflecting cultural norms prioritizing familial care (58–60). In contrast, a study in urban Japan found that living with children increased formal care access, possibly due to better integration of family and healthcare systems in urban settings (61, 62). The reliance on family support in our rural cohort may stem from limited healthcare infrastructure, where families often serve as the primary caregivers, reducing the need for formal support (63–66).

Although 58.7% of respondents had sustained an osteoporotic fracture, only 40.1% were receiving osteoporosis pharmacotherapy (Table 1). Logistic regression showed that treated individuals were more than five-times likelier to obtain post-fracture help than untreated peers. Conversely, perceived bone weakness, a proxy for untreated skeletal fragility, reduced the odds of help (OR 0.26, 95% CI 0.20–0.35). These findings indicate that inadequate pharmacologic coverage both heightens clinical risk and diminishes social support mobilization. However, only 40.1% of participants reported regular calcium or vitamin-D supplementation. Supplement users exhibited the same five-fold increase in receiving help, suggesting that nutritional intervention functions as a trigger for caregiver engagement. The remaining 59.9% without supplementation constitute a nutritionally vulnerable subgroup at risk for poor bone healing and prolonged disability. Activity avoidance was reported by 60.8% of the cohort and was strongly associated with care difficulty. Recent falls (53.8%) and mobility-aid use (20.2%) further underscore functional limitations (Table 2).

These findings have significant public health implications for rural China. The high burden of osteoporotic fractures and associated life impact necessitate enhanced screening and prevention programs. Socioeconomic disparities in care access highlight the need for expanded insurance coverage and efforts to address rural–urban healthcare inequities. Low policy awareness and high demand for government support call for targeted health education campaigns and increased public health investment in rural areas. The protective effect of medical interventions suggests that improving access to supplements and treatments could significantly enhance care outcomes.

This study’s strengths include its large sample size (3,600 older adult individuals) across diverse rural Chinese regions, a validated questionnaire (Cronbach’s alpha: 0.82) capturing both osteoporotic and normal fractures, and a focus on socioeconomic disparities (e.g., insurance: OR 2.18, *p* < 0.001) and policy awareness barriers (OR 0.22, *p* < 0.001), offering novel insights for health equity with rigorous statistical methods. This study has several limitations. First, its cross-sectional design restricts the ability to infer causality between exposures and outcomes. Second, reliance on self-reported data may introduce recall bias, particularly regarding fracture history and supplement use. Third, although the rural focus strengthens relevance for underserved populations, it limits generalizability to urban settings. Additionally, the absence of detailed fracture characteristics (e.g., anatomical site, severity) and treatment information (e.g., type, duration, adherence) constrains the ability to examine their differential impacts on post-fracture care outcomes.

We also excluded individuals with severe comorbidities and secondary osteoporosis to minimize heterogeneity in baseline health and functional status, enabling clearer estimation of associations with care-related outcomes such as help received, task difficulty, and life impact. However, this may reduce the external validity of our findings for older adult populations with complex medical profiles. Individuals with cancer-related bone disease, corticosteroid-induced osteoporosis, or end-stage organ failure often follow distinct care pathways—frequently involving institutional or hospital-based services—that differ from the community-based eldercare context examined in this study. Including such cases could have introduced confounding and obscured relationships specific to the primary osteoporosis population in rural settings. Future research should explicitly address the care needs of these medically vulnerable subgroups.

Finally, the study did not assess individual health literacy levels, which may influence understanding of policy entitlements, nor did it account for regional disparities in healthcare infrastructure across provinces. These factors could affect both policy awareness and access to support services, potentially contributing to variation in care outcomes across different rural settings.

## 5 Conclusion

This study underscores the substantial burden of osteoporotic fractures and their profound impact on the daily lives of older adult individuals in rural China, highlighting significant challenges exacerbated by socioeconomic disparities in care access, limited policy awareness, and an expressed need for enhanced governmental support. The protective role of medical interventions, such as supplements and osteoporosis treatment, suggests that improving access to these resources could substantially enhance care outcomes. These findings also advocate targeted public health strategies, including expanded screening, improved insurance coverage, and strengthened health education initiatives, to address fracture-related morbidity and care inequities in rural settings.

## Data Availability

The original contributions presented in the study are included in the article/supplementary material, further inquiries can be directed to the corresponding author.
