# Correction: Health outcomes and care needs after osteoporotic fractures in rural Chinese older adults: policy implications

**DOI:** 10.3389/fpubh.2025.1660552

**Published:** 2025-08-29

**Authors:** Qian Zhu, Caixia Ran

**Affiliations:** Department of Orthopedics and Traumatology, The Central Hospital of Enshi Tujia and Miao Autonomous, Hubei, China

**Keywords:** osteoporotic fractures, older adult care, rural China, socioeconomic disparities, policy awareness, family support

In the published article, there was an error in the legends for Table 1 and Table 2 as published. The captions erroneously stated the study population as “*N* = 3,660”. The corrected legend appears below.

Table 1. Characteristics of study participants in rural China (*N* = 3,600).

Table 2. Characteristics of study participants by received help after fracture, life impact, fracture history, and osteoporosis treatment in rural China (*N* = 3,600).

The original article has been updated.

